# Immunmodulation durch Ernährung bei kritisch kranken Patienten

**DOI:** 10.1007/s00101-023-01258-4

**Published:** 2023-02-16

**Authors:** Simon Hirschberger, Annika Schmid, Simone Kreth

**Affiliations:** 1 Klinik für Anaesthesiologie, LMU Klinikum München, München, Deutschland; 2 Walter-Brendel-Zentrum für experimentelle Medizin, Ludwig-Maximilians-Universität München (LMU), Marchioninistr. 68, 81377 München, Deutschland

**Keywords:** Immunonutrition, Immunmetabolische Paralyse, Mikrobiom, Makronährstoffe, Ketogene Diät, Immunonutrition, Immunometabolic paralysis, Microbiome, Macronutrients, Ketogenic diet

## Abstract

Kritisch kranke Patienten leiden häufig unter einer komplexen und schwerwiegenden immunologischen Dysfunktion. Die Differenzierung und Funktion von Immunzellen werden maßgeblich durch metabolische Prozesse gesteuert. Neue immunonutritive Konzepte versuchen daher, die Immunfunktionen intensivmedizinischer Patienten über enterale und parenterale Ernährung positiv zu beeinflussen. Die vorliegende Übersichtsarbeit präsentiert kondensiert die verfügbare Evidenz zu den gängigen isolierten Supplementen (antioxidative Substanzen, Aminosäuren, essenzielle Fettsäuren) und die damit verbundenen Problematiken. Im zweiten Teil werden sich daraus ergebende neuartige und umfassendere Konzepte der Immunonutrition zur Beeinflussung des intestinalen Mikrobioms und zur Modulation der Makronährstoffkomposition vorgestellt. Die Immunonutrition des kritisch kranken Patienten hat enormes Potenzial und kann sich zukünftig zu einem wertvollen klinischen Tool zur Modulation des Immunmetabolismus intensivmedizinischer Patienten entwickeln.

## Abstract (en)

Critically ill patients often suffer from a complex and severe immunological dysfunction. The differentiation and function of human immune cells are fundamentally controlled through metabolic processes. New concepts of immunonutrition therefore try to use enteral and parenteral nutrition to positively impact on the immune function of intensive care unit patients. This review article concisely presents the currently available evidence on the commonly used isolated supplements (anti-oxidative substances, amino acids, essential fatty acids) and difficulties related to their clinical use. The second part presents new and more comprehensive concepts of immunonutrition to influence the intestinal microbiome and to modulate the macronutrient composition. Immunonutrition of critically ill patients bears enormous potential and could become a valuable clinical tool for modulation of the immunometabolism of intensive care unit patients.

Der kritisch kranke Patient zeichnet sich in der Mehrzahl der Fälle durch eine schwere immunologische Fehlregulation aus [52, 192, 214]. Sowohl exzessive hyperinflammatorische Prozesse als auch eine protrahierte Immunsuppression bis hin zur Immunparalyse sind beschrieben [96, 204]. Diese intrinsische Kompromittierung des Immunsystems wird als eine entscheidende Determinante des klinischen Outcomes kritisch kranker Patienten betrachtet [29].

## Hintergrund

Das menschliche Immunsystem wird funktionell maßgeblich durch metabolische Prozesse gesteuert [137]. Das aufstrebende Forschungsfeld des Immunmetabolismus erbrachte bislang nicht nur detaillierte Kenntnisse hinsichtlich des deutlich unterschiedlichen Bedarfs verschiedener Immunzellsubpopulationen an Nährstoffen und Energieträgern, sondern konnte v. a. aufschlüsseln, dass durch das verfügbare Angebot an Metaboliten auch die Differenzierung und Funktion von Immunzellen entscheidend beeinflusst werden können [31, 60, 151]. Die Untrennbarkeit von Metabolismus und Immunologie ist von pathophysiologisch großer Relevanz. Dies manifestiert sich insbesondere in der schwersten Erschöpfung zentraler Stoffwechselprozesse in Immunzellen kritisch kranker Patienten [38]. Das Prinzip der Immunparalyse kann in diesem Zusammenhang zu einer immunmetabolischen Paralyse erweitert werden.

Die Beeinflussung des menschlichen Immunsystems bei intensivmedizinischen Patienten war und ist Gegenstand zahlreicher Forschungsprojekte. Durch die sich erweiternden Kenntnisse der engen Verbindungen zwischen Immunologie und Metabolismus sind in den letzten Jahren zunehmend nutritive Gesichtspunkte in den Fokus geraten. Es wurde versucht, über Ernährungsintervention oder -supplementation – die sog. Immunonutrition – therapeutischen Einfluss auf die Immundysregulation zu nehmen. Immunonutrition wird definiert als Ernährungsintervention mit dem Ziel einer Modulation des Immunsystems [33]. Bislang wird Immunonutrition als eine additive Therapie betrachtet, d. h. die Zugabe beispielsweise einzelner Vitamine, Amino- oder Fettsäuren zur klassischen enteralen oder parenteralen Ernährung. Durch eine supranormale Dosierung dieser Substanzen oberhalb des täglichen Bedarfs soll eine Verbesserung der immunologischen Funktion erreicht werden. In den gängigen Konzepten werden die immunologischen Auswirkungen der Zusammensetzung der Makronährstoffe noch nicht adressiert.

**Figure 1:**
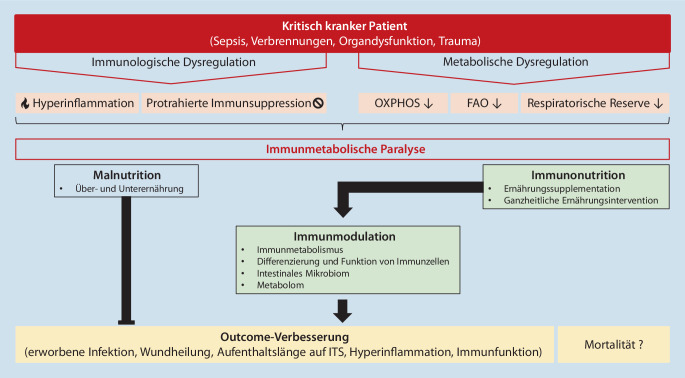

In dieser Übersichtsarbeit sollen die gegenwärtigen Kenntnisse über die Möglichkeiten der Immunmodulation durch Ernährung bei kritisch kranken Patienten zusammengefasst werden. Dabei fokussiert der erste Teil komprimiert auf die klassischen Supplemente hinsichtlich der Rationale ihres Einsatzes und der dazugehörigen Datenlage. Im zweiten Teil wird ein Blick auf neue Konzepte gewagt, und – ausgehend von den Limitationen der gegenwärtigen Evidenz und der Problematik der optimalen Supplementierungs- und Messstrategien – werden innovative Ansätze einer immunologisch wirksamen Ernährungstherapie des kritisch kranken Patienten entwickelt (Abb. 1).

## Klassische Immunonutrition

Der Versuch der Einflussnahme auf das menschliche Immunsystem durch Ernährungsinterventionen wird seit vielen Jahren wissenschaftlich untersucht. Im Kontext des intensivmedizinischen Patienten basieren die eingesetzten und in klinischen Studien untersuchten Konzepte auf der Supplementation von 1) antioxidativen und antiinflammatorischen Substanzen, 2) Aminosäuren oder 3) essenziellen Fettsäuren [54]. Diese Substanzen werden – einzeln oder in Kombination – zusätzlich zur enteralen oder zur parenteralen Ernährung appliziert. Im Folgenden werden die pathophysiologischen Grundlagen sowie die verfügbare Evidenz hinsichtlich der einzelnen immunonutritiven Konzepte vorgestellt.

### Antioxidative Substanzen

#### Pathophysiologische Grundlagen und allgemeine Evidenzlage zur Supplementation

Im Rahmen kritischer Erkrankungen zeigt sich ein deutlicher Anstieg von radikalen Sauerstoffspezies („reactive oxygen species“, ROS), die bei Erschöpfung der antioxidativen Systeme oxidativen Stress ausüben und biochemische Prozesse inhibieren, Proteine, Lipide und DNA schädigen sowie zu Zell- und schließlich Organschäden führen können [21, 22]. Eine verminderte antioxidative Kapazität bei Intensivpatienten korreliert mit einer Verstärkung der systemischen Entzündungsreaktion und einer daraus resultierenden erhöhten Morbidität und Mortalität [5, 14, 70, 163, 200]. Zusätzlich verstärken exazerbierte ROS die bereits bestehende Immundysregulation dieser Patienten [224]. Die nutritive Supplementation von antioxidativen Substanzen soll nicht nur Schäden durch oxidativen Stress minimieren, sondern auch die immunologische Homöostase fördern. Vor allem die Vitamine E und C (Letzteres nachfolgend detailliert beschrieben), Kupfer, Selen und Zink üben antioxidative und antiinflammatorische Funktionen aus und sind enteral oder parenteral als antioxidative Mikronährstoffe zuführbar [21, 35].

Über das respiratorische System, das Phosphatpuffersystem und die exogene Zufuhr von Pufferlösungen bestehen mehrere leistungsstarke Möglichkeiten der iatrogenen Einflussnahme auf das Redoxsystem abseits einer Ernährungstherapie. Aufgrund fehlender validierter Messmethoden ist unklar, ob die zusätzliche, nutritive Zufuhr antioxidativer Substanzen einen relevanten Einfluss auf das oxidative Gleichgewicht ausüben kann [73, 80]. Während eine ältere Metaanalyse positive Effekte auf die 28-Tages-Mortalität beschreibt, konnte in neueren Metaanalysen bislang kein signifikanter Effekt antioxidativer Mikronährstoffe auf die Mortalität oder die Intensivstationsverweildauer identifiziert werden [74, 90, 203]. In einer kürzlich erschienenen Metaanalyse waren unterschiedliche Kombinationen von Mikronährstoffen jeweils mit positiven Effekten auf die Mortalität, das Infektionsrisiko und den Krankenhausaufenthalt von Intensivpatienten assoziiert [75]. Zudem scheint das Ansprechen je nach Subgruppe und Substanz zu divergieren. Demnach profitieren Verbrennungs- und Traumapatienten hinsichtlich systemischer Inflammation und Infektionsanfälligkeit von der antioxidativen Wirkung einer Selen- und Zinksupplementierung [23, 25].

##### Fazit.

Die erhebliche Heterogenität der Studien und Ergebnisse rechtfertigt bislang keine allgemeine Empfehlung für den Einsatz antioxidativer Substanzen in supranormalen Dosierungen bei kritisch kranken Patienten [107]. Die Leitlinie der Deutschen Gesellschaft für Ernährungsmedizin (DGEM) empfiehlt keine routinemäßige Supplementation antioxidativer Mikronährstoffe [57]. Die Leitlinie zur Ernährung in der Intensivmedizin der European Society for Clinical Nutrition and Metabolism (ESPEN) empfiehlt antioxidative Substanzen nur zum Ausgleich eines bewiesenen Mangels [180]. Die „Mikronährstoff-Leitlinie“ der ESPEN empfiehlt ebenso die Selen- und Vitamin-E-Gabe bei entsprechendem Mangel und zudem die Supplementation von Zink bei Patienten mit schweren Verbrennungen [24].

#### Vitamin C

Die Supplementation von Vitamin C wurde in zahlreichen klinischen Studien untersucht, insbesondere bei septischen Patienten. Vitamin C gilt als pleiotrope Substanz, die neben einer antioxidativen Kapazität verschiedene Funktionen des Immunsystems beeinflusst [36]. Die i.v.-Supplementation von Vitamin C erfolgte in den verschiedenen Untersuchungen in stark unterschiedlichen Dosierungen sowie in Mono- und in Kombinationstherapie, insbesondere zusammen mit Thiamin und Hydrocortison. Diese Heterogenität wirkt sich auch auf die bisherige Evidenzlage aus. Mehrere Metaanalysen haben keine Reduktion der Mortalität kritisch kranker Patienten unter Vitamin-C-Gabe aufzeigen können [119, 174, 215]. Andere aktuelle Metaanalysen geben Hinweise auf einen möglichen dosisabhängigen Effekt von Vitamin C. In den Subgruppen der hochdosierten Gabe von Vitamin C (6–25 g/Tag) haben sich signifikante Assoziationen zu einer verminderten Mortalität gezeigt [61, 156, 225]. In weiteren systematischen Übersichtsarbeiten ergaben sich unter Vitamin-C-Supplementation Hinweise auf eine Verbesserung des Sequential Organ Failure Assessment (SOFA) Score sowie Assoziationen zu einem Rückgang der erforderlichen Dosis an Vasopressoren, ohne Einfluss auf die Mortalität der Patienten [13, 66, 172, 222].

Die Heterogenität der Studienlage spiegelt sich auch in den derzeit gültigen Leitlinien: Während die DGEM- und die ESPEN-Leitlinien zur Ernährung in der Intensivmedizin keine Empfehlung zum Einsatz von Vitamin C geben, spricht sich die aktuelle „Mikronährstoff-Leitlinie“ der ESPEN für die Gabe von 2–3 g/Tag während der akuten inflammatorischen Phase einer kritischen Erkrankung aus [24, 57, 180].

Für erhebliche Kontroverse sorgten die Daten einer neuen großen randomisierten Studie über den Einsatz von Vitamin C bei septischen Patienten: Die hochdosierte Gabe von 50 mg/kgKG Vitamin C alle 6 h führte dazu, dass der gemeinsame primäre Endpunkt aus Mortalität und Organdysfunktion signifikant häufiger erreicht wurde. Für die isolierten Komponenten des gemeinsamen Endpunkts (Mortalität oder Organdysfunktion) waren die Steigerungen unter Vitamin-C-Supplementation hingegen nicht signifikant [109]. Eine Vielzahl an jüngst publizierten randomisierten kontrollierten Studien hat ebenfalls – an sehr viel kleineren Patientenpopulationen – die Hochdosisgabe von Vitamin C untersucht. Teilweise sind signifikante Effekte auf sekundäre Endpunkte (weniger Vasopressorenbedarf, verbesserte Oxygenierungsleistung, Abnahme inflammatorischer Biomarker, Zunahme der Nierenersatztherapie) berichtet worden. Hinsichtlich der Mortalität, der Intensiv- oder Krankenhausverweildauer zeigten sich keine signifikanten Unterschiede zur Placebogruppe [95, 104, 124, 170, 205, 223].

##### Fazit.

Die Heterogenität der bisherigen Ergebnisse könnte ein Indikator dafür sein, dass bestimmte Subgruppen – beispielsweise Patienten mit besonders ausgeprägtem Vitamin-C-Mangel – von hochdosierter Vitamin-C-Gabe profitieren könnten [186]. Bis weitere Studien die Datengrundlage erweitern, unterstützt die aktuelle Evidenz bislang noch nicht die routinemäßige Anwendung von Vitamin C bei septischen Patienten [160, 186, 218].

### Aminosäuren

#### Glutamin

Glutamin ist eine semiessenzielle Aminosäure. In Stresssituationen, wie beispielsweise während intensivmedizinischer Therapie, ist die endogene Synthese nicht bedarfsdeckend, da Glutamin für zahlreiche biochemische und insbesondere auch immunologische Prozesse benötigt wird [45]. Die Rationale hinter dem immunonutritiven Einsatz von Glutamin ist seine Bedeutung als wichtiger Nährstoff für Immunzellen sowie als Vorstufe des zentralen Antioxidationssystems Glutathion [72, 189]. Eine geringe Serum-Glutamin-Konzentration bei Aufnahme auf die Intensivstation ist mit dem Schweregrad der Erkrankung assoziiert, und ein Glutaminmangel gilt als unabhängiger Risikofaktor, der mit einem schlechteren Outcome der Patienten korreliert [27, 32, 49, 212]. Der immunmetabolische Einfluss von Glutamin ist komplex und übersteigt die Rolle als bloßer „Nährstoff“. Glutamin verstärkt die Differenzierung, Aktivierung und Immunantwort von proinflammatorischen T‑Helferzellen der Typen 1 und 17 (Th1- und Th17-Zelen), wohingegen regulatorische T‑Zellen supprimiert werden [98, 106, 143, 179]. Glutamin ist zudem der Ausgangsstoff der succinatinduzierten Interleukin(IL)-1β-Inflammationskaskade in Makrophagen [194]. Eine deutlich erhöhte Zufuhr von Glutamin könnte den kritisch kranken Patienten immunologisch durch eine übermäßige Stärkung proinflammatorischer Prozesse weiter kompromittieren. Entsprechend sind auch erhöhte Glutaminspiegel ein unabhängiger Risikofaktor für Mortalität und korrelieren mit einem schlechteren intensivmedizinischen Outcome [181].

In zahlreichen Studien bei intensivmedizinischen Patienten unter mechanischer Beatmung zeigte sich ein allenfalls geringer Benefit oder sogar eine erhöhte Mortalität durch Glutaminsupplementation [83, 86, 211, 221]. In mehreren Metaanalysen wurden übereinstimmend eine Reduktion der Intensivstationsverweildauer und eine Abnahme (nosokomialer) Infektionen durch Glutamingabe festgestellt; dies bleibt ohne signifikanten Effekt auf die Mortalität der Patienten [7, 39, 141, 150, 185, 191, 220]. Bei Verbrennungspatienten hingegen erbrachten 2 Metaanalysen eine signifikante Reduktion der Mortalität unter Glutaminsupplementation [116, 220]. In einer jüngst veröffentlichten großen randomisierten kontrollierten Studie an über 1200 Verbrennungspatienten konnte hingegen kein positiver Effekt von Glutamin auf die Krankenhausverweildauer und die Mortalität detektiert werden [85].

##### Fazit.

Auf Basis der verfügbaren Evidenz wird bislang eine generelle enterale Glutaminsupplementationstherapie nicht empfohlen, die ESPEN Leitlinie spricht sich aber für den Einsatz bei kritisch kranken Verbrennungspatienten aus [56, 180].

#### Arginin

Arginin ist als ebenfalls konditionell-essenzielle Aminosäure an einer Vielzahl biochemischer Prozesse beteiligt; diese sind im Rahmen der Immunantwort, der Entzündungsreaktion, der Wundheilung und der Energiebereitstellung von Bedeutung [219]. Insbesondere ist Arginin für die Funktion und Proliferation von T‑Zellen unabdingbar [147, 168, 193]. Im Rahmen der Sepsis ist ein stark erhöhter Argininbedarf bei limitierter endogener Synthese beschrieben [12], und auch nach größeren Operationen findet sich eine Suppression der Argininspiegel und infolgedessen der T‑Zell-Funktion [198]. Prominent ist die Rolle von Arginin als Vorstufe des Stickstoffmonoxids (NO), dem zentralen Regulator von Gefäßtonus und -permeabilität [17, 64, 130]. Zudem soll NO über die Regulation von endothelialen Adhäsionsmolekülen, Zytokinen und Transkriptionsfaktoren antiinflammatorische Effekte ausüben [46, 59, 183, 184, 206]. Entsprechend war der Einsatz von Arginin beim kritisch kranken und hämodynamisch instabilen Patienten stets auch von einer gewissen Kontroverse hinsichtlich evtl. sich verschlechternder Kreislaufparameter begleitet [87, 122, 155, 188].

Die verfügbare Evidenz zur Supplementation von Arginin als Immunmodulator beim kritisch Kranken ist ausgesprochen limitiert. Besonders kritisch ist der regelhafte Einsatz anderer Nährstoffsupplemente wie Fettsäuren, Antioxidanzien oder Nukleotiden in Kombination mit Arginin, sodass die verschiedenen Studien nur eingeschränkt miteinander verglichen werden können. Eine Argininsupplementation hat in der Zusammenschau der heterogenen Studienlage möglicherweise einen positiven Effekt auf das Auftreten von sekundären Infektionen und die Krankenhausverweildauer, aber keinen signifikanten Einfluss auf die Mortalität bei Intensivpatienten [18, 51, 63, 82, 84, 102, 129, 139, 155].

##### Fazit.

Aufgrund der Heterogenität der Studien und der untersuchten Patienten (intensivmedizinisch vs. perioperativ) sowie des Mangels an Daten zur isolierten Gabe von Arginin beim kritisch kranken Patienten wird die Supplementation von Arginin bei intensivmedizinischen Patienten derzeit nicht empfohlen [56, 133].

### Fettsäuren

Mehrfach ungesättigte Omega-3(ω3)- und Omega-6(ω6)-Fettsäuren („polyunsaturated fatty acids“, PUFA) stellen über ihre Metaboliten Eikosapentaen‑/Dokosahexansäure (EPA/DHA; ω3) und Arachidonsäure (ω6) essenzielle Grundbausteine für pro- und antiinflammatorische Mediatoren (insbesondere Prostaglandine und Leukotriene) dar. Da beide Stoffwechselwege um dieselben Enzyme konkurrieren, kann durch Verschiebung des Gleichgewichtes in Richtung ω3-PUFA eine verhältnismäßige Zunahme der Synthese antiinflammatorischer Metaboliten generiert werden [16].

Sowohl die Gabe von ω3-PUFA als auch die Supplementation von Fischöl als Lieferant insbesondere der ω3-Fettsäuren DHA und EPA bei kritisch kranken Patienten soll zur Abmilderung hyperinflammatorischer Zustände führen. Für nahezu alle Immunzellpopulationen ist ein inhibierender Einfluss durch EPA/DHA nachgewiesen worden [76]. Entsprechend könnte sich gleichzeitig auch das Risiko erhöhen, eine bereits bestehende Immunsuppression zu aggravieren.

Die verfügbaren Metaanalysen zum Einsatz von Fischöl oder ω3-PUFA bei kritisch kranken Patienten konnten bisher keine Verbesserung der Mortalität nachweisen [108, 111, 114, 127, 152]. Die in neueren Veröffentlichungen untersuchten Daten zeigen aber 1) unter parenteraler Supplementation von ω3-PUFA eine signifikante Abnahme von Infektionen, Intensivstations- und Krankenhausverweildauer, 2) unter ω6-reduzierender parenteraler Lipidgabe ebenfalls eine Abnahme der Krankenhausverweildauer sowie 3) in der Subgruppe der isolierten Fischölsupplementation eine signifikante Abnahme der 28-Tages-Mortalität kritisch kranker Patienten [145, 161].

#### Fazit.

Die ESPEN-Leitlinie gibt an, dass eine parenterale Supplementation von ω3-PUFA bei kritisch kranken Patienten erfolgen kann, während die DGEM-Leitlinie bislang von einer Empfehlung absieht [56, 180].

### Nukleotide

Obgleich an nahezu allen zellulären Vorgängen beteiligt, werden Nukleotide bei Notwendigkeit einer beschleunigten Zellproliferation lediglich als konditionell essenziell betrachtet. Im Rahmen intensivmedizinischer Therapie soll durch die exogene Zufuhr von Nukleotiden die hohe Zellteilungsrate von Immunzellen unterstützt werden [132]. Entsprechend konnte grundlagenwissenschaftlich die elementare Bedeutung von Nukleotiden für die Differenzierung, Proliferation und Funktion zahlreicher Immunzellpopulationen nachgewiesen werden [81]. Abseits dieser Überlegungen fehlen bislang Studien, die den Einsatz von Nukleotiden als Immunonutrition beim Erwachsenen und insbesondere beim kritisch kranken Patienten untersuchen.

#### Fazit.

Aufgrund der fehlenden Datenlage kann keine Empfehlung zum supplementären Einsatz von Nukleotiden ausgesprochen werden.

### Gründe für die heterogene Evidenz zur Wirksamkeit

Die klassische Immunonutrition hat aufgrund der heterogenen Evidenz einer Wirksamkeit bislang keinen einheitlichen Eingang in die ernährungsmedizinischen Empfehlungen und Leitlinien für intensivmedizinische Patienten gefunden [56]. Ein möglicher Grund liegt in der Heterogenität der bisherigen Studien. Die große Variabilität der eingesetzten Dosierungen und die variierenden Kombinationen verschiedener Supplemente erschweren die Kondensierung der einzelnen Datensätze.

Eine zweite Problematik ergibt sich aus der zeitlichen Dynamik der kritischen Erkrankung. In der S2k-Leitlinie („Klinische Ernährung in der Intensivmedizin“) der DGEM wird bereits auf die grundlegende Beachtung der Akut- und Postakutphase für die Ernährungstherapie bei intensivmedizinischen Patienten hingewiesen [56]. In Bezug auf die immunologische Situation der Patienten erscheint die Lage noch komplexer: Entgegen früheren Annahmen treten Hyperinflammation und Immunsuppression nicht geordnet in einem biphasischen Verlauf auf. Vielmehr liegt eine komplexe Immundysregulation vor, mit dynamisch ineinander übergehenden oder parallel auftretenden hyperinflammatorischen und immunparalytischen Phasen [91, 214]. Somit ist – ähnlich der Schwierigkeit des Studiendesigns bei immunologisch wirksamen Arzneimitteln – die Frage ungeklärt, wann welcher Patient welche Form der Immunmodulation benötigt. Ohne Kenntnis des individuellen immunologischen Status der Patienten ist eine isoliert pro- oder antiinflammatorische Immunonutrition mutmaßlich nicht zielführend.

Ein dritter Erklärungsansatz findet sich in der Betrachtung des systemischen Stoffwechsels von intensivmedizinischen Patienten. Die massenspektrometrische Analyse des Metaboloms ermöglicht eine umfassende Erfassung und Quantifizierung von Metaboliten im menschlichen Serum. Diese Untersuchungen haben aufzeigen können, dass sich das menschliche Metabolom z. B. im Rahmen der Sepsis fundamental ändert [110]. In Anbetracht dessen stellt sich die Frage, ob selbst die hochdosierte Supplementation eines einzelnen – grundlegend immunmodulatorisch wirksamen – Nahrungsbestandteils überhaupt einen relevanten biologischen Effekt und klinischen Nutzen ausüben kann.

## Mögliche zukünftige Konzepte der Immunmodulation

Im Folgenden werden neue, innovative Ansätze einer immunologischen Ernährungsintervention vorgestellt; diese wirken nicht isoliert pro- oder antiinflammatorisch, sondern zielen darauf ab, eine Immunhomöostase wiederherzustellen. Die erste verfügbare Evidenz soll zusammengefasst und diese Immunonutrition auch unter dem Blickwinkel der oben beschriebenen Problemfelder bewertet werden.

### Mikrobiom und Immunonutrition

#### Gegenwärtige Kenntnisse und bisherige Strategien

**Figure 2:**
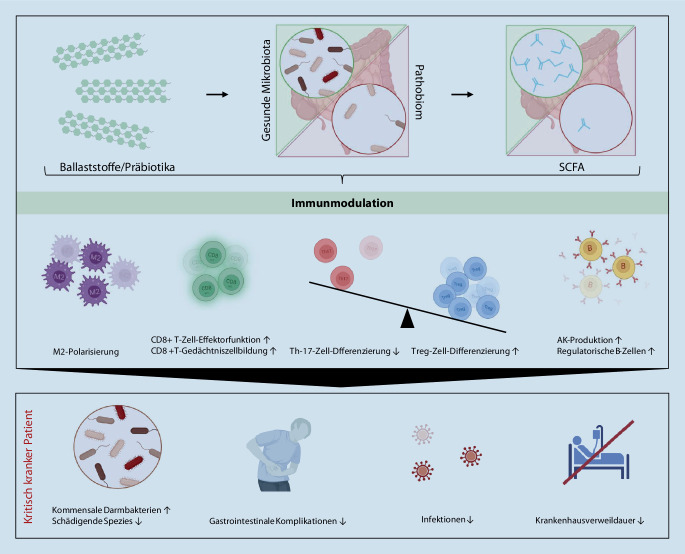

Die Bedeutung des Darmmikrobioms für die menschliche Gesundheit ist in den letzten Jahren zunehmend wissenschaftlich untersucht worden. Die zentrale Bedeutung der intestinalen Bakterienflora für den Metabolismus und die Immunologie des Menschen ist mittlerweile fest etabliert [2]. Entsprechend werden Störungen des mikrobiellen Milieus (Dysbiose) mit zahlreichen Krankheitszuständen in Verbindung gebracht [195]. Über nutritive Faktoren besteht die Möglichkeit einer fundamentalen Einflussnahme auf die Zusammensetzung des Mikrobioms [226]: Veränderungen in der Zusammensetzung der Makronährstoffe wirken sich bereits innerhalb von 24 h in gravierender Form aus [213]. Auch bei intensivmedizinischen Patienten kann z. B. die Zusammensetzung der intestinalen Flora durch die Gabe von Ballaststoffen beeinflusst werden [126]. Dadurch entwickelt sich das Darmmikrobiom zu einer wichtigen und spannenden Stellschraube in der Immunonutrition (Abb. 2).

Das Darmmikrobiom stellt durch eine Hemmung der Kolonisation humanpathogener Keime bereits einen eigenen „First-line“-Schutz gegen oral-pathogene Keime dar [19]. Das intestinale Epithel vermittelt einerseits Homöostase und Toleranz gegenüber der kommensalen Flora und beteiligt sich andererseits sowohl direkt als auch über die Aktivierung und Steuerung einer (adaptiven) Immunantwort an der Abwehr pathogener Noxen [4, 158].

Eine entscheidende immunologische Rolle nehmen kommensale Bakterien über ihre metabolischen Interaktionen mit dem menschlichen Organismus ein. Durch die Produktion und Verstoffwechselung von Fetten, Gallensäuren und Tryptophan wirken sie mittelbar auf zahlreiche Immunzellpopulation [20, 136]. Aus der Fermentierung nichtresorbierbarer Kohlenhydrate – zugeführt als (lösliche) Ballaststoffe – erzeugen Darmbakterien kurzkettige Fettsäuren („short chain fatty acids“, SCFA). Diese stellen nicht nur das primäre Substrat zur Energieversorgung von Enterozyten dar [167], sondern üben weitreichenden Einfluss auf den humanen Immunmetabolismus aus [202]. Die SCFA stimulieren oxidative Stoffwechselwege in Makrophagen und polarisieren diese in Richtung des antiinflammatorischen M2-Phänotyps [175]. In humanen CD8-T-Zellen zeigen sich nach gesteigerter SCFA-Synthese im Rahmen einer ballaststoffreichen Diät eine verbesserte antivirale Effektorfunktion und eine vermehrte Gedächtniszellbildung, bedingt durch einen verstärkten Fettsäuremetabolismus und eine gesteigerte mitochondriale oxidative Phosphorylierung [15, 197]. Über die Produktion von SCFA und die Umsetzung von Gallensäurenmetaboliten werden die Th17-Zell-Differenzierung und die IL-17A-Synthese durch die intestinale Flora supprimiert, während gleichzeitig die Differenzierung, Proliferation und Funktion von (auch intestinalen) regulatorischen T‑Zellen (Treg) gestärkt werden [53, 62, 77, 154, 182]. Für B‑Zellen ist durch SCFA sowohl eine verstärkte Antikörperproduktion als auch kompensatorisch die Induktion regulatorischer B‑Zellen beschrieben worden [105, 171]. Diese Metaboliten dienen somit intestinal und systemisch einer Stärkung sowohl pro- als auch antiinflammatorischer Immunzellpopulationen, womit sie über die Regulierung der (adaptiven) Immunität direkten Einfluss auf die immunologische Homöostase ausüben.

Auch für die Intensivmedizin hat das intestinale Mikrobiom eine fundamentale Bedeutung. Kritische Erkrankungen per se – noch ohne antibiotische Therapie – führen bereits nach wenigen Stunden zu einer erheblichen Abnahme der bakteriellen Besiedlung (1/1000). Dieses sog. Pathobiom ist neben einer „ultra-low diversity“ zudem von einer Zunahme pathogener und inflammationsassoziierter Bakterien sowie einer deutlichen Abnahme der SCFA-produzierenden Bakterien geprägt [3, 79, 146]. Entsprechend ist eine geringe Diversität des Mikrobioms auch mit einer längeren intensivmedizinischen Behandlung assoziiert [1].

Diese Situation wird durch den regelhaften Einsatz von Antibiotika bei kritisch kranken Patienten weiter aggraviert. Auch die – sowohl durch bakterizide wie auch durch bakteriostatische – Antibiotika induzierte Dysbiose ist gekennzeichnet durch einen massiven Rückgang der Anzahl und Diversität der intestinalen Flora, verminderte metabolische Aktivität sowie eine relative Zunahme pathogener Keime [125, 148, 165]. Dies hat eine schwere intestinale immunologische Dysregulation zur Folge, mit Dominanz proinflammatorischer Makrophagen und exzessiver Th1-Immunantwort [175]. Gleichzeitig führt die antibiotikainduzierte Dysbiose aber auch durch den Wegfall der gegenseitigen Signalmechanismen zu einer Kompromittierung der angeborenen und adaptiven Immunantwort [20]. Diese Veränderungen treten unmittelbar nach einer Antibiotikagabe auf und zeigen einen protrahierten Verlauf, mit teilweise über Jahre anhaltenden residuellen Veränderungen [48, 199].

Entsprechend könnte die Stärkung des intestinalen Mikrobioms einen wichtigen Ansatzpunkt für zukünftige innovative Immunonutrition darstellen. Unterschieden werden im klinischen Einsatz die Gaben von Prä- und Probiotika. Präbiotika sind (lösliche) Kohlenhydratballaststoffe, die von Darmbakterien verstoffwechselt werden. Durch die Anreicherung dieser Nährstoffe sollen Wachstum und Funktion der kommensalen Flora unterstützt werden. Der Begriff Probiotika hingegen bezeichnet lebende Mikroorganismen, die als Supplement zugeführt werden. Dadurch soll versucht werden, als vorteilhaft geltende kommensale Darmbakterien anzureichern und mittelbar schädigende Spezies zu supprimieren.

Die in zahlreichen Metaanalysen zusammengefasste Evidenz hinsichtlich des Einsatzes von Probiotika zeigt bei limitierter Studienqualität eine Reduktion an sekundären Infektionen und beatmungsassoziierten Pneumonien, aber keinen Effekt im Hinblick auf die Intensivstationsverweildauer oder die Mortalität der Patienten [28, 37, 97, 128, 177, 190, 208, 210]. Auch in den neuesten prospektiven randomisierten Studien konnte kein Effekt einer Probiotikagabe auf Mortalität, nosokomiale Infektionen oder den Intensivstationsaufenthalt nachgewiesen werden [99, 118, 207]. Die immunonutritive Supplementation von Probiotika basiert bislang auf der Gabe einzelner oder weniger Spezies. Möglicherweise lässt sich dadurch die Komplexität des Mikrobioms und somit auch der theoretisch positive immunmetabolische Effekt nur insuffizient abbilden. Entsprechend findet sich bislang in den Leitlinien keine Empfehlung zur Gabe probiotischer Supplemente bei kritisch kranken Patienten.

Die Gabe von Ballaststoffen im engeren Sinn (Präbiotika) bei kritisch kranken Patienten ist hingegen bislang nur in wenigen Studien untersucht worden. Die bei intensivmedizinischen Patienten deutlich verminderten SCFA-Level konnten durch die Zufuhr von Ballaststoffen gesteigert werden [149]. Zudem zeigten sich eine Reduktion der Raten an Diarrhöen und gastrointestinalen Komplikationen sowie Hinweise auf positive Effekte bezüglich Blutzuckerhomöostase, infektiöser Komplikationen und Krankenhausverweildauer [71]. Bislang konnten allerdings keine signifikanten Auswirkungen auf die Mortalität erfasst werden [34, 166]. Eine ähnliche Datenlage findet sich zur Gabe von Synbiotika – also dem kombinierten Einsatz von Pro- und Präbiotika – welche eine Abnahme nosokomialer Infektionen bewirken, aber ohne Auswirkungen auf die Mortalität [115, 128, 178].

In der Vergangenheit existierten aufgrund des Risikos einer möglichen Darmischämie keine einheitlichen Empfehlungen zur Gabe von Ballaststoffen. Gemäß neuerer Studien und Metaanalysen ist hingegen der Einsatz von Ballaststoffen bei kritisch kranken Patienten – die als hämodynamisch ausreichend stabil für eine enterale Ernährung betrachtet werden – als sicher zu bewerten [71]. Entsprechend wurde der mögliche Einsatz von Ballaststoffen in der DGEM-Leitlinie aufgeführt [56]. Weitere experimentelle und klinische Studien hinsichtlich der immunologischen und klinischen Auswirkungen einer präbiotischen Immunonutrition sind somit wünschenswert.

#### Potenzieller Ansatzpunkt einer neuen Interventionsmöglichkeit

Da mutmaßlich ein erheblicher Teil der positiven immunologischen Effekte der intestinalen Flora indirekt über SCFA vermittelt werden, könnte deren direkte Supplementation einen alternativen Ansatz darstellen. Tatsächlich moduliert die Gabe von Butyrat oder Propionat in Tierversuchen und auch in ersten humanen Studien die Zusammensetzung der intestinalen Mikrobiota und vermittelt antiinflammatorische Effekte [43, 58, 65, 135, 157]. Um den Stellenwert der direkten Verabreichung von SCFA gegenüber der indirekten Anreicherung durch Pro- oder Präbiotika zu beurteilen, werden dringend weitere humane Studien benötigt, die den Einsatz von SCFA als Immunonutrition auch speziell bei kritisch kranken Patienten untersuchen.

### Effekte von Makronährstoffen auf das Immunsystem des Menschen

In Anbetracht der fundamentalen Änderungen des Metaboloms kritisch kranker Patienten sind die biologischen Effekte der isolierten Supplementation von Einzelsubstanzen vielleicht insuffizient. Um einen signifikanten Einfluss auf das menschliche Immunsystem zu erreichen, muss Immunonutrition umfassender gedacht werden. Möglicherweise ist es erforderlich, auch die Grundlagen der Ernährungstherapie kritisch zu hinterfragen.

Bislang basiert die verfügbare enterale und parenterale Sondenkost in der Zusammensetzung ihrer Makronährstoffe auf den für die gesundheitliche Prävention der Allgemeinbevölkerung gedachten Empfehlungen der Deutschen Gesellschaft für Ernährung (DGE). Dies entspricht einem Verhältnis Kohlenhydrate:Fette:Proteine von ungefähr 55:30:15 Energie-%. Dabei fehlt bislang die wissenschaftliche Evidenz für eine derartige Verteilung der Makronährstoffe, und die (limitierten) verfügbaren klinischen Daten sind bestenfalls kontrovers [47, 176].

Klar ist, dass Kohlenhydrate, Fette und Proteine weitreichende immunologische und metabolische Auswirkungen haben können [153]. Die erhebliche Bedeutung der Makronährstoffe per se für das menschliche Immunsystem ist bislang insbesondere im Kontext der als schädlich geltenden „Western Diet“ untersucht worden [40, 42, 121, 144]. Im nachfolgenden Teil werden die bisherigen Kenntnisse über die immunologischen Folgen der einzelnen Makronährstoffe zusammengefasst, und es wird ein Ausblick über neue Möglichkeiten einer Immunonutrition gegeben.

#### Gegenwärtige Kenntnisse und bisherige Strategien

##### Proteine.

Die Bedeutung von Aminosäuren auf das menschliche Immunsystem wurde im Rahmen der Supplementation von Arginin und Glutamin bereits skizziert. Insbesondere die Synthese von Zytokinen und Antikörpern basiert auf einem ausreichenden Angebot von aminosäureliefernden Proteinen [196]. Dies ist bei kritisch Kranken mit entsprechend hohem „turn-over“ insbesondere von Akute-Phase-Proteinen enorm wichtig [92, 169]. Darüber hinaus erfüllen Aminosäuren wichtige Funktionen im Rahmen der Aktivierung, Proliferation und Effektorfunktion zahlreicher Lymphozyten (T-Zellen, B‑Zellen, Makrophagen, natürliche Killer[NK]-Zellen, [117]). Verzweigtkettige Aminosäuren werden im Speziellen mit einer verstärkten Differenzierung von Treg-Zellen in Verbindung gebracht [94]. In der Betrachtung der Immunonutrition stand bislang die Supplementation einzelner Aminosäuren im Vordergrund. Es ist noch unklar, welche Menge und welche Zusammensetzung an Proteinen oder Aminosäuren einen supportiven immunologischen Effekt ausüben kann.

##### Fettsäuren.

Die mögliche Bedeutung von ω3- und ω6-Fettsäuren (PUFA) auf das Gleichgewicht pro- und antiinflammatorischer Mediatoren wurde bereits ausführlich beschrieben. Allgemein stellen FA einen elementaren Energielieferanten und Bestandteil von Zellmembranen dar. Ihre ubiquitäre Rolle im menschlichen Metabolismus umfasst die Regulation der Genexpression und zahlreicher Zellfunktionen [196]. Als integrale Bestandteile der Zellmembran auch von Immunzellen sowie als Lieferant von Energie und Wegweiser der metabolischen Ausrichtung der Zellen beeinflussen FA die Immunzellfunktion, beispielsweise die T‑Zell-Aktivierung und -differenzierung [26, 93, 173]. Fettsäuren werden über die β‑Oxidation der mitochondrialen oxidativen Phosphorylierung zugeführt. Diese ist von zentraler Bedeutung für die immunologische Kompetenz und Grundlage der essenziellen Funktion der Mitochondrien für das menschliche Immunsystem [101, 137]. Bislang ist unklar, welche Effekte direkt auf FA oder indirekt auf einen gesteigerten mitochondrialen Metabolismus zu attribuieren sind und inwieweit die isolierte Modulation der Fettzufuhr positive immunologische Effekte vermitteln kann.

##### Kohlenhydrate.

Die Zufuhr von Kohlenhydraten wird mitunter als essenziell bezeichnet. Tatsächlich jedoch ist der menschliche Organismus auf die exogene Zufuhr von Kohlenhydraten nicht angewiesen. Selbst unter extremer Kohlenhydratrestriktion ist über die hepatische Glukoneogenese ein ausreichendes Substratangebot für obligat glykolytische Zellen, wie Erythrozyten oder das Nebennierenmark, gegeben [89]. Neben nachteiligen metabolischen Effekten sind mittlerweile aber auch die negativen immunologischen Folgen einer primär kohlenhydrathaltigen Ernährung umfassend charakterisiert worden [41]. Über die glucoseinduzierte Aktivierung des „NLR-family-pyrin-domain-containing-3“(NLRP3)-Inflammasoms wird eine chronische „Low-grade“-Inflammation initiiert, unterhalten und über insulinabhängige Mechanismen weiter verstärkt [40, 50]. Durch hohe Glucosezufuhr und konsekutiv hohe Insulinkonzentrationen entsteht ein immunmetabolischer „Teufelskreis“. Dieser mündet langfristig in multiplen Organdysfunktionen, in der Promotion und Aggravation zivilisatorischer Erkrankungen und in einer Schwächung des adaptiven Immunsystems [42]. Die Bedeutung der inflammatorischen Wirkung von insbesondere nichtkomplexen Kohlenhydraten zeigt sich exemplarisch darin, dass bereits die einmalige Zufuhr von Nahrung mit einem hohen glykämischen Index die Serumkonzentrationen des C‑reaktiven Proteins (CRP) und von proinflammatorischen Proteinen signifikant ansteigen lässt [120, 138].

Entsprechend konnten in einer Vielzahl von Studien die vorteilhaften Effekte einer Kohlenhydratrestriktion auf das Immunsystem charakterisiert werden. Diese umfassen eine reduzierte unspezifische Inflammation (bei Gesunden und auch bei Kranken) bei erhaltener oder sogar gesteigerter immunologischer Effektorfunktion auf einen spezifischen Reiz wie beispielsweise eine Infektion oder Impfung [44, 100, 134, 142, 216]. Auf zellulärer Ebene wird dies auf eine metabolische Umprogrammierung von Glykolyse hin zur Nutzung von Fettsäuren im Rahmen der mitochondrialen oxidativen Phosphorylierung zurückgeführt [112].

#### Kohlenhydratrestriktion als potenziell neue Möglichkeit der Immunonutrition

**Figure 3:**
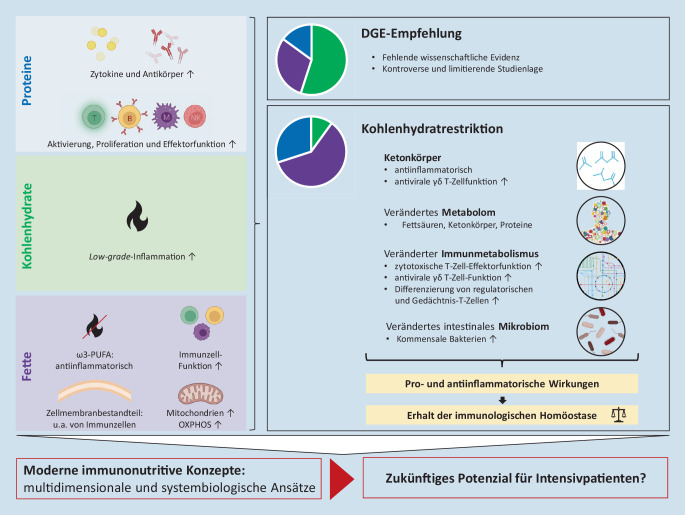

Geht man davon aus, dass Kohlenhydrate immunologisch nachteilige, Fettsäuren hingegen eher supportive Effekte ausüben, könnte durch die grundlegende Veränderung der Zusammensetzung der Makronährstoffe ein neuer Ansatzpunkt für eine zukünftige klinische Immunonutrition entwickelt werden (Abb. 3). Bei einer Restriktion der Kohlenhydratzufuhr und entsprechender Steigerung des nutritiven Anteils an FA wird die endogene hepatische Synthese von Ketonkörpern initiiert [162]. Eine derartige isokalorische Ernährung entspricht in der Zusammensetzung der Makronährstoffe einem Verhältnis Kohlenhydrate:Fette:Proteine von ungefähr 10:60:30 Energie-% und wird als „ketogene Diät“ bezeichnet.

Die immunologischen Auswirkungen einer ketogenen Diät sind umfassend. Ketonkörper wirken antiinflammatorisch und können durch Inhibition des NLRP3-Inflammasoms chronisch Low-grade-Entzündungen supprimieren [67, 217]. Gleichzeitig wird die antivirale Immunantwort von γδ T-Zellen durch eine ketogene Diät verstärkt, sodass bereits der Einsatz von Ketonkörpern als antivirale Therapieoption postuliert wurde [68, 69, 187]. Das menschliche adaptive Immunsystem reagiert auf eine isokalorische Kohlenhydratrestriktion mit einer grundlegenden immunmetabolischen Reprogrammierung der T‑Zellen in Richtung aerober mitochondrialer Energiegewinnung. Dies bewirkt eine umfassende Stärkung insbesondere der zytotoxischen Effektorfunktion der T‑Lymphozyten und steigert gleichzeitig die Differenzierung von regulatorischen und Gedächtnis-T-Zellen [88, 89, 103]. Im klinisch-translationalen Setting konnten bereits die fundamentale Bedeutung des ketogenen Metabolismus für menschliche T‑Zellen im Kontext des kritisch kranken COVID-19-Patienten dargestellt und zudem insbesondere die Verbesserung des T‑Zell-Immunmetabolismus durch Ketonkörper auch bei kompromittierten T‑Zellen von intensivmedizinischen Patienten nachgewiesen werden [88, 103].

Durch die gravierend veränderte Zusammensetzung der Makronährstoffe hat eine ketogene Diät auch erhebliche Auswirkungen auf das Metabolom und das intestinale Mikrobiom, wodurch mittelbar zusätzliche modulierende Einflüsse auf Immunzellpopulationen ausgeübt werden [6, 131].

Eine isokalorische kohlenhydratreduzierte Makronährstoffmodulation wirkt in mehrfacher Hinsicht auf das menschliche Immunsystem: 1) direkt immunologisch über Ketonkörper, 2) über Stoffwechselintermediate und ein verändertes Metabolom, 3) über den zellulären Immunmetabolismus und 4) über die kommensale Darmflora. Das additive Zusammenspiel dieser Wirkmechanismen könnte ein Erklärungsansatz für die deutliche Steigerung der immunologischen Kompetenz durch eine ketogene Diät sein. Ein weiterer entscheidender Vorteil liegt im Erhalt der immunologischen Homöostase aufgrund der Augmentierung sowohl pro- als auch antiinflammatorischer Mechanismen [89].

Damit zeigt eine ketogene Ernährungsintervention auf, wie moderne immunonutritive Konzepte zukünftig über multidimensionale und systembiologische Ansätze zentrale Schwachstellen der bisherigen Supplemente kompensieren könnten. Im klinischen Einsatz hat eine ketogene Diät zusätzlich den Vorteil, dass sie lediglich über den Austausch der enteralen Nährstofflösung erfolgt und somit denkbar einfach umzusetzen ist. Entsprechend ist eine erste klinische Studie zur Evaluation der Auswirkungen einer ketogenen Ernährung auf septische Intensivpatienten bereits abgeschlossen [164]. Die Publikation der Ergebnisse wird in sehr naher Zukunft erwartet.

## Schlussbetrachtungen

Grundvoraussetzung für jede Form von Immunonutrition ist die Vermeidung von Malnutrition. Eine adäquate Immunantwort benötigt eine erhebliche Zufuhr von Kalorien und Proteinen, um ihren enormen bioenergetischen Bedarf zu decken [30]. Ohne die basale Abdeckung des Energie- und Nährstoffbedarfs ist eine darüber hinausgehende Immunonutrition wenig sinnvoll [209].

Tatsächlich leidet ein Großteil der intensivmedizinischen Patienten unter einer Mangelernährung [113]. Diese korreliert mit einem verlängerten Aufenthalt auf der Intensivstation, protrahierter maschineller Beatmung, vermehrten Infektionen und einer erhöhten Mortalität [55, 123, 159, 201]. Eine adäquate Erfassung des Ernährungsstatus, die Identifikation etwaiger Risiken einer Mangelernährung, eine sinnvolle diagnostische Überwachung und kontrollierte Ernährungstherapie sind also insbesondere aufgrund des per definitionem kritischen Ernährungszustandes von intensivmedizinischen Patienten von fundamentaler Bedeutung und für das Outcome des kritisch kranken Patienten essenziell. Über diese Basisparameter hinaus jedoch ist die Umsetzung von ernährungsmedizinischen Protokollen und Standards auf Intensivstationen weiterhin ausbaufähig [87, 140].

Die klinische Ernährungsmedizin ist ein sich entwickelndes Gebiet. Die geringe Standardisierung von Ernährungsinterventionen und die fehlende Aussagekraft ausgewerteter Fragebogen zur retrospektiven Erfassung des Diätverhaltens von Probanden oder Patienten limitieren bislang die Evidenzlage und den medizinischen Einfluss von Ernährungsinterventionen erheblich [8, 10, 11].

Die nutritive Therapie von kritisch kranken Patienten hat den entscheidenden Vorteil, dass durch iatrogene Zuteilung der Nahrung tatsächlich eine standardisierte und überwachbare Ernährungstherapie durchgeführt werden kann. Klinische Ernährungstherapie basiert nicht auf „Anekdoten einer Diät“ [9], sondern ermöglicht auf valider Datengrundlage die Generierung der notwendigen Evidenz. Dadurch kann erreicht werden, dass Ernährung nicht nur als „notwendiges Übel“ zur bedarfsdeckenden Energieversorgung der Patienten betrachtet wird, sondern dass Ernährungsinterventionen als innovatives und wertvolles klinisches Tool verstanden werden, um den Immunmetabolismus der kritisch kranken Patienten positiv zu beeinflussen.

## Fazit für die Praxis

- Für die klassischen immunonutritiven Supplemente (Antioxidanzien, Vitamin C, Arginin, Glutamin, Fettsäuren, Nukleotide) gibt es teilweise Hinweise auf eine Reduktion sekundärer Infektionen sowie der Intensivstationsverweildauer, bislang aber nur heterogene Evidenz bezüglich des Rückgangs der Mortalität in Subgruppen. Ihr routinemäßiger Einsatz bei kritisch kranken Patienten wird bislang nicht einheitlich empfohlen.
- Aufgrund der komplexen immunologischen Dysregulation und der fundamentalen Beeinflussung des Metaboloms intensivmedizinischer Patienten sind wahrscheinlich umfassendere immunonutritive Konzepte erforderlich.
- Die Beeinflussung des intestinalen Mikrobioms über Prä- und Probiotika ermöglicht eine Stärkung der kommensalen Flora und – über Metaboliten wie Short chain fatty acids (SCFA) – weitreichende Effekte auf humane Immunzellpopulationen und könnte somit zukünftig – klinische Evidenz vorausgesetzt – eine Rolle in der Immunonutrition kritisch Kranker einnehmen.
- Die Modulation der Makronährstoffkomposition zugunsten von Fetten (Kohlenhydratrestriktion) kann über die Produktion von Ketonkörpern, über den zellulären Immunmetabolismus sowie über ein verändertes Metabolom und Mikrobiom umfassend auf die immunologische Homöostase einwirken. Dieses Wissen birgt für die Intensivmedizin zukünftig ein großes klinisches Potenzial.
- Durch eine kontrollierte iatrogene Nährstoffzuteilung ermöglicht intensivmedizinische Immunonutrition die Generierung der erforderlichen Evidenz, um Ernährungsinterventionen zu einem klinischen Tool zur Beeinflussung des menschlichen Immunmetabolismus entwickeln zu können.
